# Analysis of risk factors for unplanned reoperation following primary repair of gastrointestinal disorders in neonates

**DOI:** 10.1186/s12871-021-01345-2

**Published:** 2021-04-23

**Authors:** Yu Cui, Rong Cao, Jia Li, Ling-mei Deng

**Affiliations:** Department of Anesthesiology, The Affiliated Hospital, School of Medicine, UESTC Chengdu Women’s & Children’s Central Hospital, No.1617, Riyue Avenue, Qingyang District, Chengdu, 610091 P.R. China

**Keywords:** Intraoperative hypothermia, Unplanned reoperation, Neonates, Gastrointestinal disorders

## Abstract

**Background:**

The aim of our study was to identify the factors associated with unplanned reoperations among neonates who had undergone primary repair of gastrointestinal disorders.

**Methods:**

A retrospective chart review was conducted for neonates who underwent primary gastrointestinal surgery between July 2018 and September 2020. The neonates were divided into two cohort, depending on whether they had an unplanned reoperation. The primary outcome was the occurrence of unplanned reoperation. The risk factors that associated the occurrence of unplanned reoperation were examined.

**Main results:**

Two hundred ninety-six neonates fulfilled the eligibility criteria. The incidence of unplanned reoperation was 9.8%. Analyses of all patients with respect of developing unplanned reoperation showed that the length of operative time was an independent risk factor [Odds Ratio 1.02; 95% confidence interval 1.00, 1.04; *p* = 0.03]. Patients with unplanned reoperation had a longer postoperative hospital length-of-stay [19.9 ± 14.7 vs. 44.1 ± 32.1 days; p<0.01].

**Conclusion:**

The current study is the first analysis of risk factors associated with an unplanned reoperation in neonates undergoing primary repair of gastrointestinal disorders. The length of operative time is the only risk factor for an unplanned reoperation, and the unplanned reoperation can directly prolong the postoperative hospital length-of-stay.

**Trial registration:**

This study was registered at http://www.chictr.org.cn/index.aspx with No. ChiCTR2000040260.

## Background

Surgical intervention is typically an effective procedure in neonates with gastrointestinal disorders, such as congenital anorectal malformation, hypertrophic pyloric stenosis, congenital intestinal atresia, and neonatal necrotizing enterocolitis. It is well understood that compared to children and adults, neonatal patients are at higher risk in the perioperative period [1]. With the development of remarkable healthcare advances in neonatal surgery, most patients have excellent outcomes after surgical interventions, but some neonates with postoperative complications still require unplanned reoperation.

According to the prior publications, rates of unplanned abdominal reoperation varied from 14 to 38.8% in neonates with jejunoileal atresia [2–5]. In 2019, Zhu et al. presented that 15 (6.0%) of neonates required reoperation following Ladd’s procedure [6]. Meanwhile, they revealed that the incidence of unplanned reoperation was 17.9% in neonates with apple-peel atresia following surgical intervention [7]. The indications for unplanned reoperation in neonates with gastrointestinal disorders included surgical site infections, functional obstruction [8], operative adhesive intestinal obstruction and anastomotic obstruction [7].

In other fields, the unplanned reoperation rate had been used as an indicator for evaluating a hospital’s ability to provide safe and efficient care [9]. Unplanned reoperations that associated with prolonged length of hospital stay and raised healthcare costs increased medical burden. Moreover, unplanned reoperations decreased patients’ satisfaction and strongly associated with surgical and medical complications [10]. Ademuyiwa et al. had clarified that reoperation was a significant determinant of mortality in neonates with intestinal obstruction [11]. However, few studies investigated the risk factors associated with unplanned reoperation following primary repair of gastrointestinal disorders in neonates.

We, therefore, reviewed our experience to identify the factors associated with unplanned reoperations among neonates who had undergone primary repair of gastrointestinal disorders. The aim of our study was to provide evidence on optimizing clinical decision-making and improving outcomes in those patients.

## Methods

This was a single center, descriptive, retrospective study, which was conducted at a large tertiary hospital (> 300 beds in NICU) in western region of China. Ethic approval was obtained from the Institutional Review Board (IRB) in Chengdu Women’s and Children’s Central Hospital. This study was registered at http://www.chictr.org.cn/index.aspx with No. ChiCTR2000040260 on November 27, 2020. The informed consent was waived because of anonymous patients’ data by the IRB of Chengdu Women’s and Children’s Central Hospital.

### Patient selection

All anesthesia and perioperative warming methods were performed in accordance with the guidelines in our institution. Neonates (aged 0 ~ 28 days) who received primary gastrointestinal surgery between July 2018 and September 2020 were enrolled in our study. Patients without sufficient data for analysis were excluded. Patients were divided into two cohorts, depending on whether they had an unplanned reoperation. An unplanned reoperation was defined as an unexpected return to the operating room within 30 days due to complications related to the original surgical intervention, regardless of whether the event occurred during a hospitalization or a readmission. The primary outcome was the occurrence of unplanned reoperation. Then, the risk factors that associated the occurrence of unplanned reoperation were identified.

### Data collection

The data was extracted from the institutional database, combining with medical chart review. Demographic variables were collected, i.e., age at surgery, weight at surgery, birth weight, gender, gestational days, ASA status, preoperative hemoglobin, and preoperative transfusion. Intraoperative outcomes were also recorded, including type of surgeries, surgical services, the length of operative time, duration of anesthesia time, mean body temperature, the incidence of hypothermia, the length of intraoperative hypothermia, estimated blood loss, blood transfusion, fluid infusion, postoperative hemoglobin, and intraoperative urinary output. Postoperative variables included postoperative ventilator dependence hours, postoperative hemoglobin, acute renal failure (AKI), postoperative hospital length-of-stay (PLOS) and hospital mortality. Duration of anesthesia time was defined as the time from anesthesia induction to discharge from the operating room at the initial operation. The length of operative time was calculated from skin incision to the end of suture. The diagnostic criteria of AKI were based on the modified KDIGO definition of neonatal AKI [12]. Hospital mortality data was obtained according to the protocol of our previous study [13]. Notably, total length of actual PLOS was calculated from surgical date to discharge date. If the patient is readmitted, PLOS need to be accumulated. The variables were double-checked by medical chart review.

### Definition of inadvertent intraoperative hypothermia

In our institution, intraoperative temperature was routinely monitored through esophagus rather than the rectum. According to the definition of the National guideline in UK [14], intraoperative hypothermia was defined as body temperature<36.0 °C. The patient’s core temperature was continuously monitored and recorded automatically at 5 min interval until discharging from the operating room. When < 36.0 °C was recorded on the electronic anesthesia record, this patient was considered to have hypothermia [14]. The length of intraoperative hypothermia was calculated from the beginning of hypothermia to the beginning of normothermia. If hypothermia occurred repeatedly, the length of hypothermia needed to be added up. All temperature data were extracted at the primary surgery.

### Statistical analysis

Continuous variables were presented as the mean ± standard deviation [SD] or median and interquartile range [IQR] (25–75%) if nonnormally distributed. The student *t* test was used to compare normally distribute data, otherwise the Mann Whitney U-test was used to compare two groups. The categorical variables were expressed as numbers and percentages. The Pearson’s chi-squared test or Fisher exact test was used as appropriate. Statistical analyses progressed from univariate to multivariate analyses (Tables 1 and 2). Univariate logistic regression was performed to identify associations with unplanned reoperation. Significant variables in univariate analysis were added to the multivariate regression model (Table 3). Clinically significant variables and those with *p*-value on univariate regression of < 0.20, were subsequently included in a multivariate regression model [15]. All analyses were conducted with R studio 3.5.2. *P* <  0.05 was statistically significant, and all tests were two-sided.

**Table 1:** Demographics and characteristics of eligible neonates

	Patients, No. (%)			
		Unplanned Reoperation		***p*** value
Variables	Total (*N* = 296)	No (*N* = 267)	Yes (*N* = 29)	
Gender, male, n (%)	177 (59.8)	161 (60.3)	16 (55.2)	0.74 ^a^
Birth weight (kg), median (IQR)	2.9 (2.2, 3.3)	2.9 (2.2, 3.3)	2.8 (2.0, 3.1)	0.30 ^b^
Very low birth weight (< 1.5 kg)	24 (8.1)	20 (7.5)	4 (13.8)	0.27 ^c^
Weight at surgery (kg), median (IQR)	2.9 (2.2, 3.3)	2.9 (2.2, 3.3)	2.9 (2.4, 3.2)	0.85 ^b^
Gestational age (days), median (IQR)	266.0 (245.0, 274.0)	266.0 (245.0, 274.0)	258.0 (244.0, 274.0)	0.49 ^b^
Gestational weeks (< 37), n (%)	127 (10.0)	112 (8.2)	15 (17.2)	0.42 ^a^
Gestational weeks (37 ≥), n (%)	169 (90.0)	155 (91.8)	14 (82.8)	
Age at the surgery (days), median (IQR)	6.0 (4.0, 12.0)	6.0 (4.0, 11.0)	6.0 (4.0, 16.0)	0.25 ^b^
ASA				0.91 ^c^
I	1 (0.3)	1 (0.4)	0 (0.0)	
II	83 (28.0)	76 (28.5)	7 (24.1)	
III	191 (64.5)	171 (64.0)	20 (69.0)	
IV	21 (7.1)	18 (7.1)	2 (6.9)	
Preoperative hemoglobin (g/dl), mean ± SD	147.2 ± 45.3	147.5 ± 45.8	144.0 ± 41.2	0.90 ^b^
Preoperative blood transfusion, n (%)	46 (15.5)	38 (14.2)	8 (27.6)	0.11 ^a^

Note: *n* Sample, *SD* Standard deviation, *IQR* Interquartile Range

^a^denotes a *p* value based on a Pearson’ chi-squared test

^b^denotes a *p* value based on a Mann-Whitney U-test

^c^denotes a *p* value based on a fisher exact test

**Table 2:** Intraoperative and postoperative data

Variables		Unplanned reoperation		***p*** value
	Total (***N*** = 296)	No (***N*** = 267)	Yes (***N*** = 29)	
Type of surgeries				0.10 ^a^
Elective, n (%)	85 (28.7)	81 (30.3)	4 (13.8)	
Emergency, n (%)	211 (71.3)	186 (69.7)	25 (86.2)	
Surgical services, n (%)				
Anoplasty	60 (20.3)	59 (22.1)	1 (3.4)	0.03* ^a^
Appendectomy	1 (0.3)	1 (0.4)	0 (0.0)	1.00 ^a^
Diaphragmatic hernia repair	4 (1.4)	4 (1.5)	0 (0.0)	1.00 ^a^
Enterectomy	194 (65.5)	168 (62.9)	26 (89.7)	< 0.01* ^b^
Gastric perforation repair	9 (3.0)	8 (3.0)	1 (3.4)	1.00 ^a^
Hernia repair	2 (0.7)	2 (0.7)	0 (0.0)	1.00 ^a^
Intestinal malrotation repair	3 (1.0)	3 (1.1)	0 (0.0)	1.00 ^a^
Intestinal Volvulus reduction	4 (1.4)	3 (1.1)	1 (3.4)	0.34 ^a^
Ovariocysrectomy	2 (0.7)	2 (0.7)	0 (0.0)	1.00 ^a^
Pyloric myotomy	5 (1.7)	5 (1.9)	0 (0.0)	1.00 ^a^
Umbilical bulge repair	12 (4.1)	12 (4.5)	0 (0.0)	0.62 ^a^
Operative time (min), mean ± SD	94.0 ± 45.6	91.3 ± 43.5	119.3 ± 58.1	< 0.01* ^c^
Operation time > 120 min, n (%)	63 (20.8)	49 (18.4)	14 (48.3)	< 0.01* ^b^
Duration of anesthesia (min), mean ± SD	164.6 ± 61.9	162.1 ± 60.7	187.3 ± 65.9	< 0.01* ^c^
Mean intraoperative temperature (°C), mean ± SD	35.8 ± 0.8	35.8 ± 0.8	35.6 ± 0.7	0.11 ^c^
≥ 36 and < 37.5 (°C), n (%)	134 (45.3)	125 (46.8)	9 (31.0)	0.15 ^b^
≥ 35 and < 36.0 (°C), n (%)	86 (29.1)	77 (28.8)	9 (31.0)	0.86 ^b^
< 35.0 (°C), n (%)	76 (25.7)	65 (25.7)	11 (37.9)	0.17 ^b^
Duration of hypothermia (min), mean ± SD	91.1 ± 83.4	87.4 ± 82.5	124.4 ± 83.0	< 0.01* ^c^
The incidence of hypothermia, n (%)	243 (82.1)	215 (80.5)	28 (96.6)	0.04* ^b^
Intraoperative blood loss (ml), mean ± SD	4.4 ± 4.0	4.2 ± 3.8	5.7 ± 5.5	0.08 ^c^
Intraoperative transfusion (ml), mean ± SD	6.7 ± 12.0	6.3 ± 11.2	10.5 ± 19.2	0.99 ^c^
Intraoperative total fluid infusion (ml/kg/h), mean ± SD	1.3 ± 0.6	1.3 ± 0.6	1.2 ± 0.5	0.38 ^c^
Intraoperative urine output (ml/kg/h), mean ± SD	1.9 ± 1.5	2.0 ± 1.5	1.7 ± 1.2	0.15 ^c^
Postoperative hemoglobin (g/dl), mean ± SD	127.3 ± 36.1	127.7 ± 36.0	123.7 ± 37.1	0.40 ^c^
Postoperative ventilation (hours), median (IQR)	35.1 (16.4, 68.7)	33.9 (16.4, 67.9)	42.3 (17.3, 81.9)	0.35 ^c^
Ventilation dependence> 48 h, n (%)	107 (36.1)	93 (34.8)	14 (48.3)	0.22 ^b^
Postoperative AKI, n (%)	27 (9.1)	25 (9.4)	2 (6.9)	0.92 ^a^
Postoperative hospital length-of-stay (days), mean ± SD	22.2 ± 17.6	19.9 ± 14.7	44.1 ± 32.1	< 0.01* ^c^
Mortality, n (%)	33 (11.1)	29 (10.9)	4 (13.8)	0.63 ^a^

Note: *n* Sample, *SD* Standard deviation, *IQR* Interquartile Range, *AKI* Acute kidney injury, *P < 0.05

^a^denotes a *p* value based on a fisher exact test

^b^denotes a *p* value based on a Pearson’ chi-squared test

^c^denotes a *p* value based on a Mann-Whitney U-test

**Table 3:** Univariate and Multivariate analysis of risk factors associated with unplanned reoperation in neonates after gastrointestinal procedures

Independent Variable	Univariate analysis		Multivariate analysis	
	OR (95% CI)	*P* value	Adjusted OR (95% CI)	*P* value
Weight at surgery (kg)	0.94 (0.55, 1.6)	0.83		
Birth weight < 1.5 (kg)	1.98 (0.63,6.24)	0.24		
Gestational weeks (< 37 vs. ≥ 37)	1.48 (0.69, 3.2)	0.32		
Type of surgeries (Elective vs. Emergency)	2.72 (0.92, 8.07)	**0.07**	0.33 (0.10, 1.09)	0.07
Surgical services, n (%)				
Anoplasty	0.13 (0.02, 0.94)	0.04*	0.04 (0.00, 1.15)	0.06
Enterectomy	5.11 (1.51, 17.31)	< 0.01*	0.20 (0.01, 2.92)	0.24
others	0.22 (0.03, 1.68)	**0.15**	0.05 (0.00, 1.47)	0.08
Mean intraoperative temperature, n (%)				
≥ 36 and < 37.5 (°C)	0.51 (0.22, 1.16)	**0.11**	0.74 (0.17, 3.22)	0.69
≥ 35 and < 36.0 (°C)	1.11 (0.48, 2.55)	0.81		
< 35.0 (°C)	1.9 (0.85, 4.23)	**0.12**	1.81 (0.52, 6.24)	0.35
Duration of hypothermia (min)	1.01 (1.00, 1.01)	0.01*	1.00 (0.99, 1.01)	0.82
The incidence of hypothermia, n (%)	6.77 (0.90, 50.91)	**0.06**	4.61 (0.42, 51.13)	0.21
Operative time (min)	1.01 (1.01, 1.02)	< 0.01*	1.02 (1.00, 1.04)	0.03*
Preoperative transfusion, n (%)	2.3 (0.95, 5.56)	**0.07**	1.44 (0.52, 4.00)	0.97
Duration of anesthesia (min)	1.00 (0.99, 1.01)	**0.08**	0.99 (0.98, 1.01)	0.48
Preoperative hemoglobin (g/dl)	1.00 (0.99, 1.01)	0.79		
Postoperative hemoglobin (g/dl)	1.00 (0.98, 1.01)	0.52		
Intraoperative blood loss (mL)	1.04 (0.98, 1.1)	**0.17**	0.99 (0.91, 1.05)	0.60
Postoperative ventilation (hours)	1.00 (1.00, 1.00)	**0.10**	1.00 (1.00, 1.00)	0.75

Note: Clinically significant variables and those with *p*-value on univariate regression of < 0.20 were subsequently included in the multivariate regression model; 95% Confidence Intervals (95% CI), *P < 0.05 is statistically significant

## Results

### Unplanned reoperation rates

**Fig. 1:**
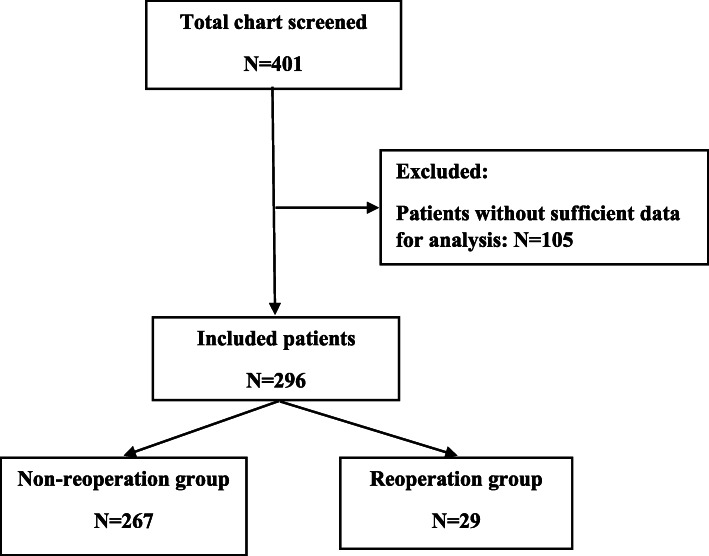
Study patient flow diagram

Overall, a total of 401 neonates undergoing gastrointestinal surgical interventions were identified between July 2018 and September 2020. Of those, 105 neonates were excluded because of missing data. Finally, 296 neonates were included in the analysis, 29 (9.8%) of them underwent an unplanned reoperation within 30 days after the initial surgery (Fig. 1).

### Demographic and clinical characteristics

Demographic and clinical characteristics for the two cohorts were presented in Table 1. More male patients (177, 59.8%) than female patients underwent gastrointestinal surgery during the neonatal period, but the difference in gender distribution between the two cohorts was not significant. There was no statistical difference between the two cohorts in terms of age, birth weight, weight at surgery, gestational days, ASA status, the value of preoperative hemoglobin or preoperative blood transfusion (Table 1). Among clinical variables, patients undergoing anoplasty were less likely to require unplanned reoperation (Non-reoperation: 59, 22.1% vs. Reoperation: 1, 3.4%; *p* = 0.03), whereas patients undergoing enterectomy were more likely to require unplanned reoperation (Non-reoperation: 168, 62.9% vs. Reoperation: 26, 89.7%; *p* <  0.01). In addition, compared to patients without reoperation, patients who underwent an unplanned reoperation had a higher incidence of intraoperative hypothermia (Non-reoperation: 215, 80.5% vs. Reoperation: 28, 96.6%; *p* < 0.01), and a longer duration of hypothermia (Non-reoperation: 87.4 ± 82.5 min vs. Reoperation: 124.4 ± 83.0 min; *p* < 0.01). Furthermore, both the length of primary operative time (Non-reoperation: 91.3 ± 43.5 min vs. Reoperation: 119.3 ± 58.1 min; *p* < 0.01) and total length of hospital stay (Non-reoperation: 19.9 ± 14.7 days vs. Reoperation: 44.1 ± 32.1 days; *p* < 0.01) were longer in patients with unplanned reoperation than patients without reoperation (Table 2).

### Risk factor analysis

The following 13 variables (type of surgeries, anoplasty, enterectomy, other surgical services, intraoperative mean body temperature, duration of hypothermia, the incidence of intraoperative hypothermia, the length of operative time, duration of anesthesia, intraoperative blood loss, and duration of postoperative ventilator dependence) were identified as risk factors for reoperation in the univariate analysis (Table 3). Multivariable logistic regression analysis (Table 3), including factors with *P* < 0.2 on univariate analysis, identified that the length of primary operative time (OR, 1.02; 95%CI, 1.00–1.04) was the independent risk factors for unplanned reoperation.

## Discussion

In our study, we used the electrical database from Chengdu Women’s and Children’s Central hospital to identify risk factors for an unplanned reoperation following primary repair of gastrointestinal disorders in neonates. We found that the length of primary operative time was the independent risk factors for unplanned reoperation and patients with unplanned reoperation had a longer PLOS.

### The importance of identification of risk factors

To our knowledge, serious postoperative complications, such as bleeding, intestinal obstruction, leakage, and infection, can lead to an unplanned reoperation. Unplanned reoperations are harmful to the patients due to repeated exposure to anesthetics and surgical pressure, especially in neonates with immature liver and kidney function. Furthermore, unplanned reoperation increases the family’s economic and psychological burden. Additionally, Ademuyiwa et al. has proved that reoperation was a significant determinant of mortality [11]. Therefore, identification of risk factors in advance is essential to improve the quality of perioperative management and provide the evidence for medical decision-making.

### The incidence of unplanned reoperation in neonates

In neonates, surgical intervention is required because of congenital malformation or acquired condition on gastrointestinal tract. Obviously, compared with adults or older children, neonates are at a higher risk due to the requirement for more delicate surgical techniques and poorer tolerance of surgical and anesthetic pressure. The incidence of unplanned reoperation varies greatly in different types of surgery. A retrospective study evaluated the unplanned reoperation rate following plastic surgery in pediatric patients and showed an overall rate of 0.8% (137/18106) within 30 days after surgery [10]. However, 23.3% (10/43) patients required unplanned reoperation after primary repair for jejunoileal atresia [8]. Theoretically, a more complex primary procedures is associated with an increased likelihood of a patient experiencing unplanned reoperation, as were the younger age. Unfortunately, there were few studies about the incidence of unplanned reoperation after gastrointestinal surgery in neonates. Studies in neonates were focused on relatively small series, most of which focus on a single procedure. Our study summarized a total of 296 newborns who underwent gastrointestinal surgery, including 11 types of surgical procedures, of which 254 (85.8%) underwent anoplasty or enterectomy. Only 1 patient (1/60, 1.7%) who underwent anoplasty experienced an unplanned reoperation, whereas 26 (26/194, 13.4%) patients who underwent enterectomy received reoperation. Totally, 9.8% neonates suffered unplanned reoperation, which was in line with previous reports.

### Risk factors associated with unplanned reoperation

In theory, demographic characteristics, surgical services, and intraoperative features, as well as postoperative complications were all related to unplanned reoperation. In univariate analysis, anoplasty (OR 0.13; *p* = 0.04), enterectomy (OR 5.11; *p* < 0.01), duration of intraoperative hypothermia (OR 1.01; *p* = 0.01), and the length of operative time (OR 1.01; *p* < 0.01) were significantly associated with unplanned reoperation. However, our multivariate regression analyses identified that the only factor associated with unplanned reoperation was prolonged operative time, which was consistent with previous research [16]. In plastic surgery, an association between increasing operative time and incidence of unplanned reoperation had been confirmed [10]. And Sangal et al. had revealed that a greater total operation time was associated with an unplanned reoperation in major operations of the head and neck [16]. These results could be interpreted as a more complex surgical procedure would be more likely to require unplanned reoperation. The prolonged operative time could be considered as a sign of procedure complexity. Reoperation resulted in a longer PLOS (Non-reoperation: 19.9 ± 14.7 days vs. Reoperation: 44.1 ± 32.1 days, *p* < 0.001). None of the remaining variables were significantly associated with unplanned reoperation in multivariate model, though some had been identified as risk factors in existing literature. A previous report which reviewed 9 nine-year experience in managing neonates with jejunoileal atresia presented that prematurity and low birth weight were associated with functional obstruction leading to reoperation [8]. However, this conclusion was drawn from a relatively small sample size, including only 43 patients undergoing enterectomy, which reduced its credibility. With the development of neonatal care and the improvement of surgical skills in recent years, the premature neonates with low birth weight could be well treated, which might decrease the requirement of surgical intervention.

Although neither duration of hypothermia nor incidence of hypothermia were identified as risk factors in multivariant analysis, their potential effects should be taken seriously. Compared to patients without reoperation, patients with reoperation had a longer duration of hypothermia and higher incidence of hypothermia during initial surgical period. Intraoperative hypothermia might increase the risk of surgical site infection [17] and even death [18]. The authors randomly assigned the patients to either normothermic or hypothermic group. They reported that surgical site infections were 19% of patients in hypothermic group and 6% of patients in the normothermic group (*P* = 0.009), and the length of hospitalization was extended by 2.6 days in the hypothermia group (*P* = 0.01) [17]. We also demonstrated that PLOS was longer in patients with reoperation than those without reoperation. Those results could be interpreted as delayed healing because of wound infections. Additionally, a meta-analysis that included 48 studies had presented that most neonates with surgical site infection had gastrointestinal and/or colorectal surgery [19]. Surgical site infection was associated with increased risk of unplanned reoperation in major head and neck surgery [16]. Thus, we speculated that gastrointestinal surgery was associated with a high risk of unplanned secondary surgery. But in China, due to the disharmonious relationship between medical workers and patients, surgical site infection might not be recorded objectively. Therefore, limited in data collection restricted further analysis in current study.

### Limitations

The limitations in our study were as follows. First, although this cohort study was conducted in medical center which was the largest neonatal surgery center in western region of China, the sample size was still insufficient for further subgroup analysis. Second, the variation of surgeons’ expertise could not be well controlled, as the evaluation system of surgeons had not been fully established in our center. Next, gastrointestinal surgeries might be a consequence of patent ductus arteriosus (PDA) [20, 21]. The stealing blood flow from aorta to pulmonary arteries via PDA might exceed the physiological compensatory mechanisms, with a consequence of decreasing organ perfusion. The available literature has proved that prolonged ductal patency was associated with higher mortality rates and several adverse outcomes, including impaired renal function and necrotizing enterocolitis (NEC) [21]. However, in our institutional database, the diagnoses, especially in emergency surgical patients, were not well-documented. Sometimes, only the diagnosis of surgical indications was recorded. Thus, it was difficult to consider PDA as a variable in the analysis, but it was an important factor that should be discussed. In the future, we will communicate with neonatologist and surgeons to improve the patient’s diagnosis record. Last, the number of variables assessed was very limited, especially variables related to baseline clinical condition and intraoperative course. There was a high risk of unexplored confounding factors. Yet, we believed that length of operation remained a good marker of a complex procedure due to either surgical or anesthesiologic factors.

## Conclusions

The current study is the first analysis of risk factors associated with an unplanned reoperation in neonates undergoing primary repair of gastrointestinal disorders**.** The length of operative time is the only risk factor for an unplanned reoperation, and the unplanned reoperation can directly prolong the PLOS. We wish this study can help to identify high-risk patients and improve clinical decision-making.

## Data Availability

The datasets generated and analyzed during the current study are not publicly available because patients’ name and admission number are included in the datasets but are available from the corresponding author on reasonable request.

## Abbreviations

OR: Odds Ratio
IRB: Institutional Review Board
CI: Confidence interval
PLOS: Postoperative hospital length-of-stay
AKI: Acute renal failure
IQR: Interquartile range
